# Brain-State-Dependent Non-Invasive Brain Stimulation and Functional Priming: A Hypothesis

**DOI:** 10.3389/fnhum.2014.00899

**Published:** 2014-11-05

**Authors:** Elena G. Sergeeva, Petra Henrich-Noack, Michał Bola, Bernhard A. Sabel

**Affiliations:** ^1^Institute of Medical Psychology, Medical Faculty, Otto-von-Guericke University of Magdeburg, Magdeburg, Germany

**Keywords:** non-invasive brain stimulation, brain-state-dependent stimulation, functional priming, neuroimaging, EEG, neurorehabilitation, closed-loop stimulation devices

The aim of using non-invasive brain stimulation techniques in neurorehabilitation is to improve neurological function by modulating brain plasticity in the specific areas of the brain.

The fundamental idea of current stimulation treatment is that it alters cortical excitability so as to enhance plasticity in subsequent perceptual or motor training. Another goal is to achieve an entrainment of brain oscillations with external currents, which are delivered at certain frequencies to improve the functions by altering physiological activity that outlasts the stimulation period [see for review, Nitsche and Paulus, 2011; Antal and Paulus, 2013].

However, such approaches often do not consider that the brain is a dynamical system with activity levels and connectivity patterns constantly changing in a highly variable, and so far non-predictable, manner. But it has been known that processing of external (e.g., visual) stimuli depends to some extent on the instantaneous state of brain networks at stimulus onset. Similarly, the effects of the stimulation depend not only on the predefined parameters but also on the state of the brain before and during the stimulation (Silvanto et al., 2007, 2008; Herrmann et al., 2013; Neuling et al., 2013). However, the translation of these findings into clinical practice was so far not realized.

In this respect a recent study by Gharabaghi et al. (2014) is of particular interest. These authors explored the possibility of using a brain-state-dependent stimulation (BSDS) approach in post-stroke patients. Here, the subjects were instructed to imagine opening a hand (without actually doing so, moreover, the patient was not capable of hand opening) in order to achieve desynchronization of beta band oscillations within the motor neural circuits. To facilitate the execution of EEG desynchronization, a contingent haptic biofeedback to the hand was provided. Transcranial magnetic pulses were then applied to the motor cortex but only if such desynchronization was achieved, as shown by concurrently recorded EEG.

Both in a healthy control volunteer and in a patient with severe hemiparesis, BSDS induced a significant increase in excitability of the motor cortex as measured by motor evoked potentials (MEP). Notably, that only BSDS evoked substantial increase of MEP amplitude, while the stimulation pulses applied without the motor-related EEG desynchronization evoked MEP amplitude decrease, though different TMS stimulation paradigms applied independent of the brain state are currently explored to improve motor function after stroke.

An important aspect of the Gharabaghi et al. study (Gharabaghi et al., 2014) is the fact that the brain stimulation was not applied *prior to* or *alternating with* motor exercise, but *during* the neurohabilitation training. This suggests that not “simple” excitability changes were involved here (when excitability is modified by TMS through the whole stimulated area independent on specific functional activity), but that additional mechanisms were involved that altered the brain’s response to the external manipulation. The authors propose that volitional modulation of brain activity with motor imagery improved susceptibility of inherent motor circuits to TMS pulses, perhaps due to voluntary depolarization of intracortical connections targeting pyramidal tract neurons and decrease of the motor cortical excitability as did motor imagery with haptic feedback alone.

Though this experiment involved only one healthy subject and one stroke patient, this finding nevertheless is novel because it may lead to new concepts of how brain stimulation may act: in order for plastic changes to emerge in a certain brain area, the central network, and external stimulation drive should be temporally and spatially related.

In line with the study from Gharabaghi et al. (2014) is the finding that the endogenous power of brain oscillations (changing with anesthesia stages) has a huge impact on the “aftereffects” of alternating current stimulation (ACS) (Sergeeva et al., 2012). Moreover, Neuling et al. (2013) demonstrated that when the timing was just right, the phase alignment of intrinsic oscillators with the external stimulation lead to an increased amplitude of the response.

The importance of the actual brain state to determine behavioral and perceptual effects of TMS and TDS was already addressed by Silvanto et al. (2008). They showed that prior manipulation of neural activation enabled TMS to selectively target populations of neurons to increase functional resolution and achieve a selective excitation of task-related areas (Silvanto et al., 2007).

Therefore, the BSDS as described by Gharabaghi et al. (2014) may permit to accurately stimulate the brain, thereby improving task performance as a function of altered excitability in the areas, which were functionally primed.

This consideration of state-dependent stimulation is novel in the context of brain current stimulation, though it follows the early theory by Sherrington (1965), and implies that the arousal of brain structures by natural tasks leads to a certain neural constellations of excitation and inhibition, which may serve as an immanent substrate for external stimuli. Since the processing of these stimuli is dependent not only on their physical properties but also on the intrinsic constitution of the stimulated system, we hypothesize that a pre-set task-primed system may show greater responsiveness in terms of better functional output to neuromodulation by brain stimulation.

The modulation of brain activity with non-invasive current stimulation has become tremendously popular. But the major concern is how to improve their precision and effectiveness. We therefore expect that future neuromodulation approaches use increasingly more fine-tuned BSDS similar to those we have witnessed most recently with optogenetic approaches (Zemelman et al., 2002): just like the light can specifically activate cells that have optogenetic sensors, the current injection patterns could be controlled to just activate or inhibit particular (primed) neuronal populations. In this manner, functional priming of certain brain areas and even groups of neurons prior or during the current/magnetic stimulation would be a possible solution to better control efficacy and safety of non-invasive brain current stimulation.

Because the state of brain networks in patients is likely to be altered, as it was observed in our laboratory in patients with visual system damage (Bola et al., 2014), the stimulation protocols known to exert certain effects in healthy subjects might not work in the same way in patients. Therefore, while it is desirable to discover general principles of priming-dependent stimulation effects in normal subjects, it might be difficult to define protocols optimal for all patients suffering from a certain condition. Rather, stimulation methods should be used in combination with neuroimaging (Fox et al., 2012), e.g., EEG or fMRI, to probe the brain state. These efforts should ultimately lead to closed-loop devices adjusting stimulation parameters automatically based on patient’s brain activity patterns.

The BSDS approach provides a basis for a novel restoration strategy. Further exploration of the mechanisms underlying BSDS, i.e., Hebbian plasticity or homeostatic metaplasticity and gating (Ziemann and Siebner, 2008) and how to prime different modes of stimulation in functional domains beyond the motor system will help to advance the field and help us pinpoint the most effective non-invasive brain stimulation protocols for neurorehabilitation and restoration.

## Conflict of Interest Statement

The authors declare that the research was conducted in the absence of any commercial or financial relationships that could be construed as a potential conflict of interest.
